# Draft Genome Sequence of the Phytopathogenic Fungus Ganoderma boninense, the Causal Agent of Basal Stem Rot Disease on Oil Palm

**DOI:** 10.1128/genomeA.00122-18

**Published:** 2018-04-26

**Authors:** Condro Utomo, Zulfikar Achmad Tanjung, Redi Aditama, Rika Fithri Nurani Buana, Antonius Dony Madu Pratomo, Reno Tryono, Tony Liwang

**Affiliations:** a Department of Biotechnology, Plant Production and Biotechnology Division, PT SMART Tbk, Bogor, Indonesia; b Section of Bioinformatics, PT SMART Tbk, Bogor, Indonesia; c Section of Microbiome Technology, PT SMART Tbk, Bogor, Indonesia; d Section of Clonal Technology, PT SMART Tbk, Bogor, Indonesia; e Section of Genetic Engineering, PT SMART Tbk, Bogor, Indonesia

## Abstract

Ganoderma boninense is the dominant fungal pathogen of basal stem rot (BSR) disease on Elaeis guineensis. We sequenced the nuclear genome of mycelia using both Illumina and Pacific Biosciences platforms for assembly of scaffolds. The draft genome comprised 79.24 Mb, 495 scaffolds, and 26,226 predicted coding sequences.

## GENOME ANNOUNCEMENT

The African oil palm, Elaeis guineensis, is the most productive oil-bearing crop around the world. This species produces 3 to 4 metric tons of mesocarp oil per hectare annually, much more than its closest competitor soybean with 1 ton/hectare/year (1). However, the production is constrained by a serious disease, basal stem rot (BSR), which is caused by the basidiomycete Ganoderma boninense (2, 3). BSR has a major economic impact on the oil palm industry in Indonesia, Malaysia, and Papua New Guinea (3, 4). The genome sequence information for this phytopathogen was generated earlier by a joint group of researchers from France and Malaysia who assembled a total of 63 Mb using Illumina and 454 platforms to identify microsatellite markers for genetic diversity studies (5). Here, we used a similar approach with Illumina and Pacific Biosciences platforms for the Indonesian strain (G3), which is geographically distinct, whereas the heterothallic character of the fungus produces high biodiversity level in the field to complement the existing genome sequence (6, 7).

The G3 strain was isolated previously from a 14-year-old oil palm tree showing severe symptoms of BSR disease in North Sumatera Province, which is an area in Indonesia where BSR is endemic (8). The isolate was identified as G. boninense based on morphological characteristics and the DNA sequence of the internal transcribed spacer (ITS) region, which has been deposited in GenBank under the accession no. MG650116 (9). The isolate was reinoculated onto oil palm seedlings and induced severe BSR symptoms (10). Freshly revived mycelia were grown in 100 ml yeast malt broth in the dark at 28°C for 14 days (11). Fungal mycelia were harvested on a layer of Whatman paper no. 1, air dried for 15 minutes, and ground into a fine powder. Fungal DNA was extracted using a GenElute plant genomic DNA miniprep kit (Sigma-Aldrich Co., St. Louis, MO, USA) according to the manufacturer’s instructions.

The genome of the G3 strain was sequenced using the Illumina Hiseq 4000 paired-end sequencing technology combined with the PacBio RS II platforms to sequence the whole-genome shotgun libraries. Initial libraries of 300 bp for Illumina and 20 kb for PacBio were prepared using a Nextera XT-library preparation kit and P6-P4 chemistry, respectively. All sequencing reads from both platforms were assembled using DBG2OLC software to produce scaffolds (12). The genome sequencing resulted in a total genome size of 79.24 Mb with 55.9% GC content, 826× sequencing coverage, 495 scaffolds, *N*_50_ scaffold length of 272.644 Kb, and a maximum scaffold size of 1,452,774 bp. In the genome, we predicted 26,226 coding sequences (CDS) using the Augustus pipeline with an average of 330 genes per Mb sequences (13).

Compared to the publicly available G. boninense genome sequence (5), our draft was bigger and may thus complement possible existing gaps or may even reconstitute problematic sequence reads. The quality of the genome sequence is reflected by the *N*_50_ scaffold length of 272.644 Kb compared to 6.116 Kb of the present genome sequence (14, 15). This improves the current G. boninense genome sequence database, which serves as a valuable resource for genomic studies analyzing the interaction of G. boninense with oil palm.

### Accession number(s).

This whole-genome shotgun project has been deposited in GenBank under the accession no. PJEW00000000.

## References

[1] Basiron Y 2007 Palm oil production through sustainable plantations. Eur J Lipid Sci Technol 109:289–295. doi:10.1002/ejlt.200600223.

[2] Turner PD 1981 Oil palm diseases and disorders. Oxford University Press, Oxford, United Kingdom.

[3] Pilotti CA 2005 Stem rots of oil palm caused by *Ganoderma boninense*: pathogen biology and epidemiology. Mycopathologia 159:129–137. doi:10.1007/s11046-004-4435-3. 15750745

[4] Aderungboye FO 1977 Disease of the oil palm. PANS 23:305–326. doi:10.1080/09670877709412457.

[5] Mercière M, Laybats A, Carasco-Lacombe C, Tan JS, Klopp C, Durand-Gasselin T, Alwee SSRS, Camus-Kulandaivelu L, Breton F 2015 Identification and development of new polymorphic microsatellite markers using genome assembly for *Fanoderma boninense*, causal agent of oil palm basal stem rot disease. Mycological Progress 14:1–11. doi:10.1007/s11557-015-1123-2.

[6] Pilotti CA, Sanderson FR, Aitken EAB 2002 Sexuality and interactions of monokaryotic and dikaryotic mycelia of *Ganoderma boninense*. Mycological Res 106:1315–1322. doi:10.1017/S0953756202006755.

[7] Cooper RM, Flood J, Rees RW 2011 *Ganoderma boninense* in oil palm plantations: current thinking on epidemiology, resistance and pathology. The Planter 87:515–526.

[8] Purba RA, Setiawati U, Susanto A, Rahmaningsih M, Yenni Y, Rahmadi HY, Nelson SPC 2015 Indonesia’s experience of developing Ganoderma tolerant/resistant oil palm planting material, p 1–23. *In* Malaysian Palm Oil Board, proceedings of the international seminar on gearing oil palm breeding and agronomy for climate change International Society for Oil Palm Breeders, Kuala Lumpur, Malaysia.

[9] Utomo C, Werner S, Niepold F, Deising HB 2005 Identification of *Ganoderma*, the causal agent of basal stem rot disease in oil palm using a molecular method. Mycopathologia 159:159–170. doi:10.1007/s11046-004-4439-z. 15750749

[10] Rohman RA 2017. Ekspresi protein tanaman kelapa sawit (Elaeis guineensis Jacq.) yang terinfeksi *Ganoderma boninense* Pat. pada fase pembibitan. Master Thesis. Bogor Agricultural University, Bogor, Indonesia.

[11] Sivakumar R 2010 Isolation, screening and optimization of production medium for thermostable laccase production from *Ganoderma* sp. Int J Eng Sci Technol 2:7133–7141.

[12] Ye C, Hill CM, Wu S, Ruan J, Ma Z 2016 DBG2OLC: efficient assembly of large genomes using long erroneous reads of the third generation sequencing technologies. Sci Rep 6:1–9. doi:10.1038/srep31900. 27573208 PMC5004134

[13] Stanke M, Tzvetkova A, Morgenstern B 2006 AUGUSTUS at EGASP: using EST, protein and genomic alignments for improved gene prediction in the human genome. Genome Biol 7:1–8. doi:10.1186/gb-2006-7-s1-s11. PMC1810548 16925833

[14] Treangen TJ, Salzberg SL 2013 Repetitive DNA and next-generation sequencing: computational challenges and solutions. Nat Rev Genet 13:36–46. doi:10.1038/nrg3117. PMC3324860 22124482

[15] Choudhuri S 2014 Bioinformatics for beginners: genes, genomes, molecular evolution, databases and analytical tools. Academic Press, New York, NY.

